# Supplementary material for: Emergence of an antigenically drifted and reassorted influenza B virus at the end of the 2024–25 influenza season
Source: mBio. 2026 Jun 22;17(8):e01198-26. doi: 10.1128/mbio.01198-26 (PMC13463853; doi:10.1128/mbio.01198-26)
Supplement: Supplementary file 1 [file mbio.01198-26-s0002.pdf]

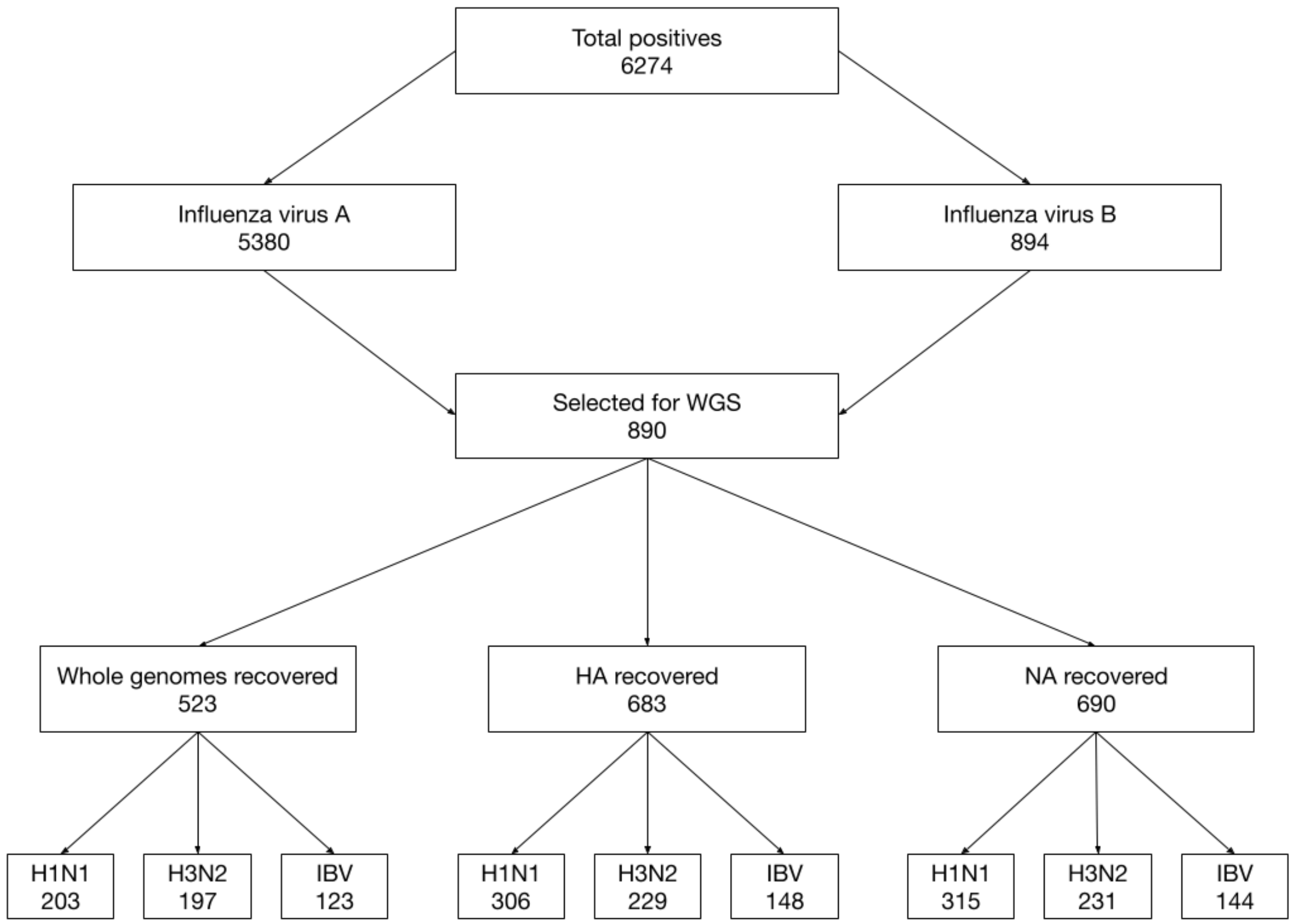

Supplemental Figure S1. Flow chart illustrating the number of samples selected for whole genome sequencing. HA and NA segments illustrate specimens where full-length segments were recovered.

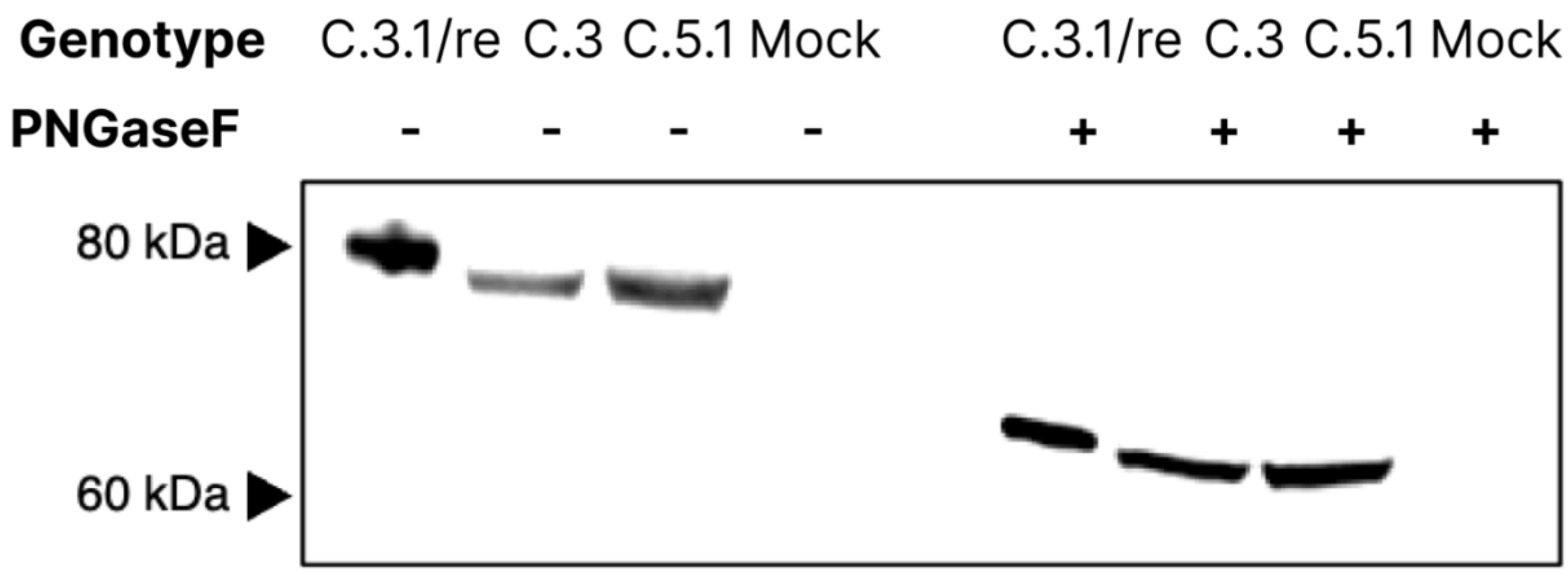

Supplemental Figure S2 – The C.3.1/re HA encodes additional N-linked glycan. Denaturing SDS PAGE gel followed by western blot of the Influenza B HA protein. MDCK-SIAT-1 cells were infected with C.3.1/re (B/Baltimore/JH-1192/2025), C.3 (B/Baltimore/JH-899/2023), or C.5.1 (B/Baltimore/JH-547/2024) and harvested after 48 hours at 33˚C. Samples were de-N-glycosylated by PNGAseF treatment and blotted in parallel.

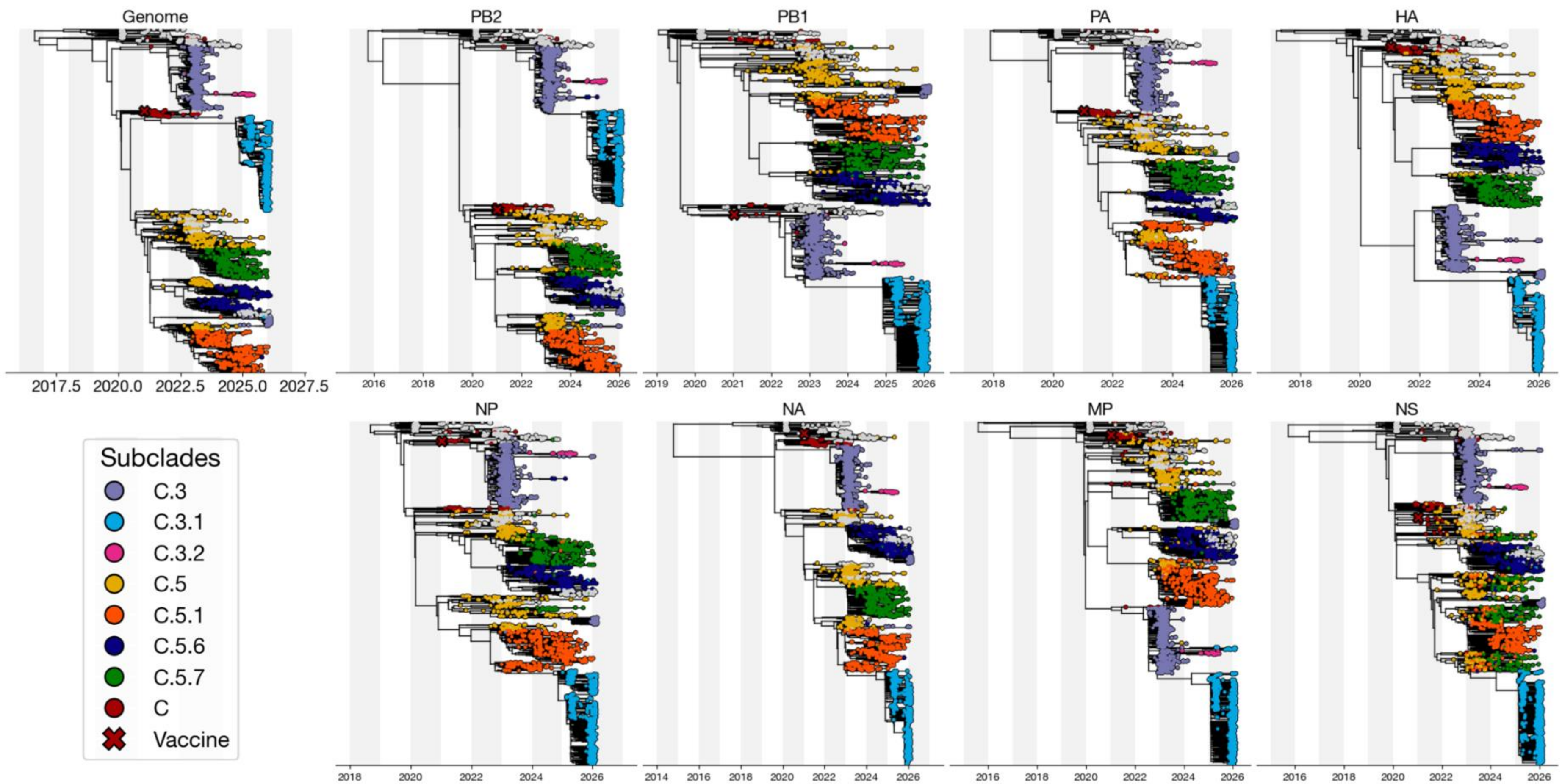

Supplemental Figure S3 – Phylogenies of the concatenated genome and 8 gene segments. The 2024-25 Influenza B vaccine component strain, B/Austria/1359417 /2021 is highlighted by an "X" on each phylogeny. Tips are colored by subclade identity as assigned by Nextclade.

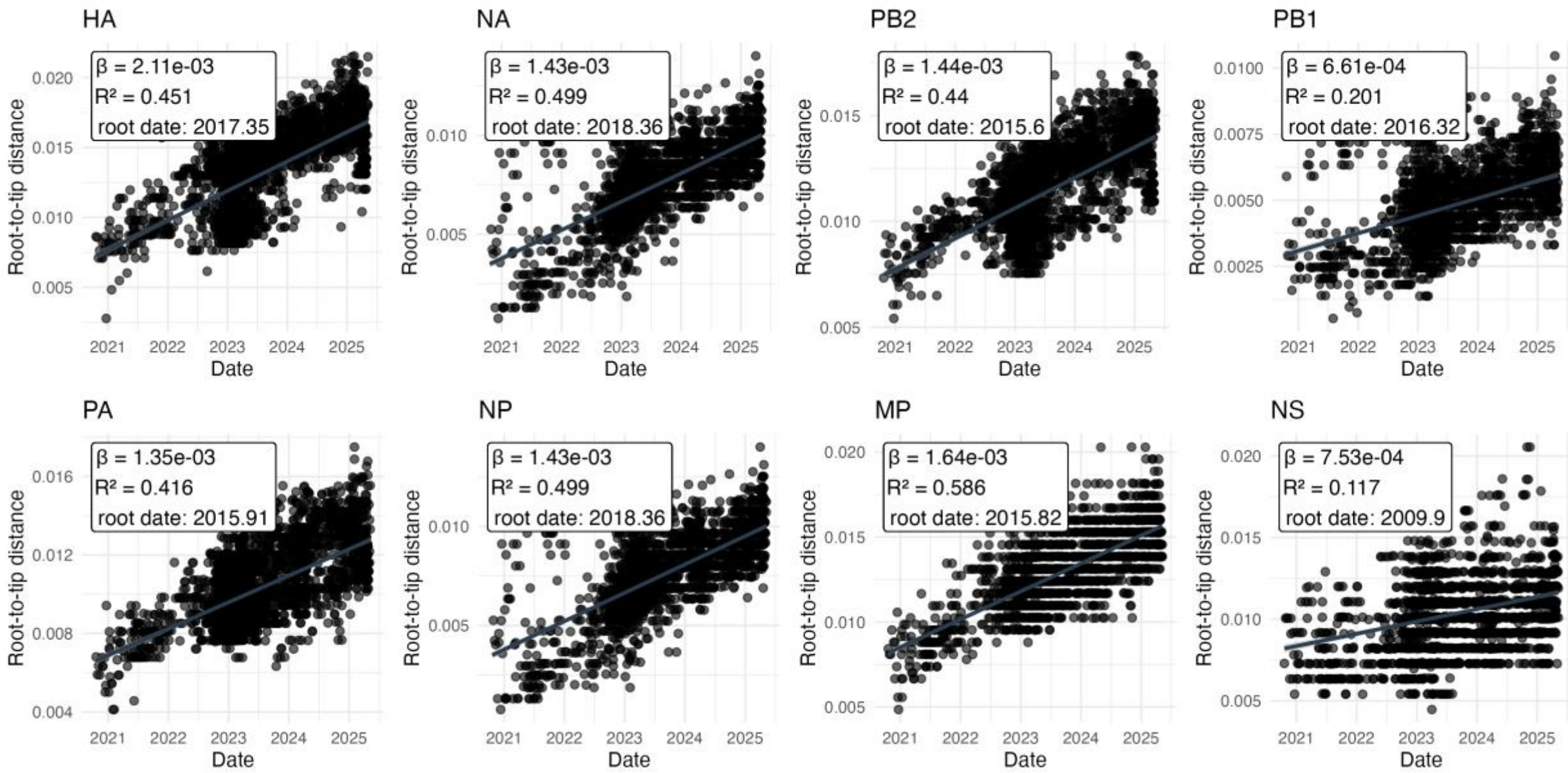

Supplemental Figure S4 – Root-to-tip regression of Influenza B segment phylogenies constructed in Treetime dated to the calculated Y intercept (root date). β represents substitutions per site per year based on either collection or sequencing date.

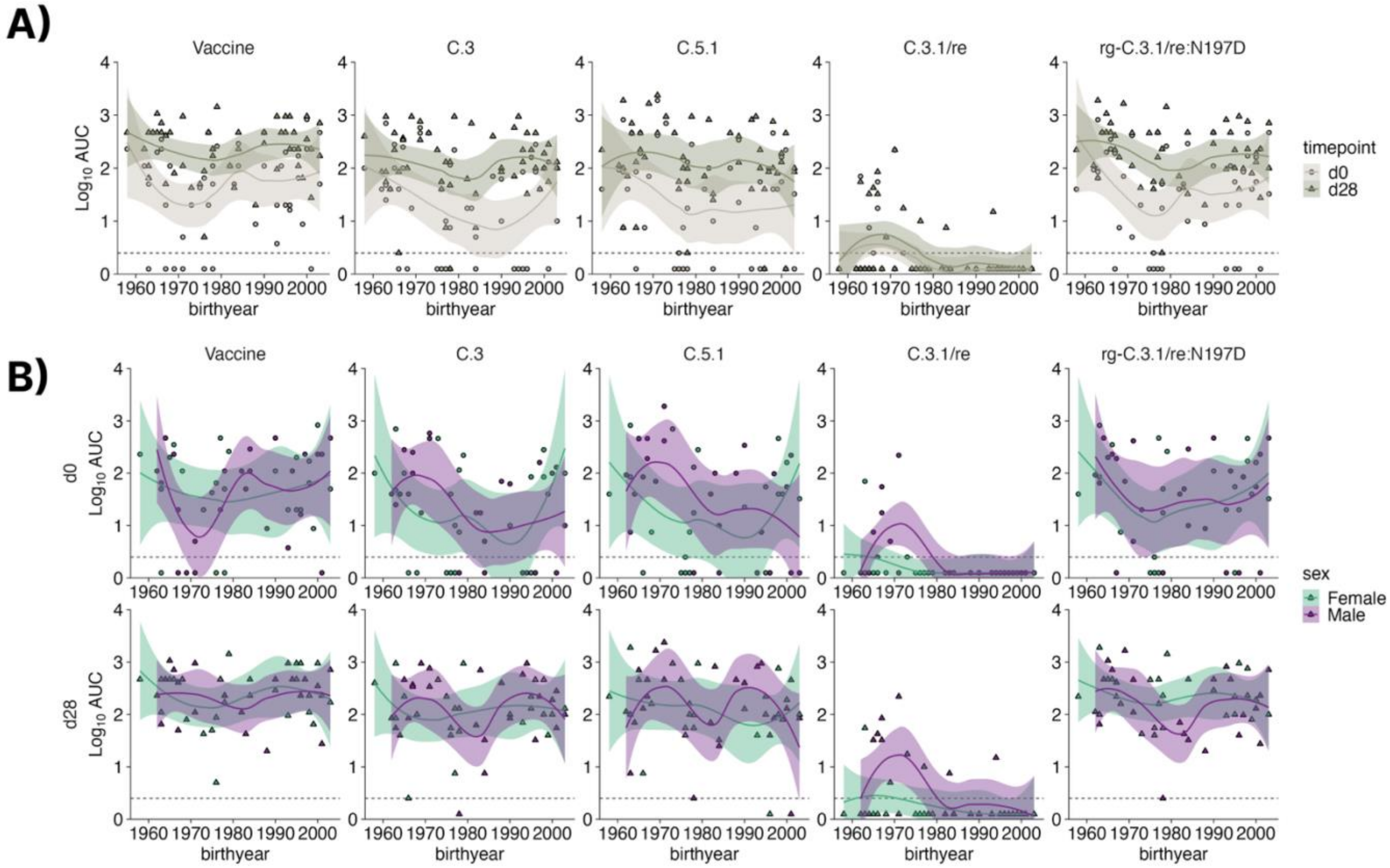

Supplemental Figure S5. Serum neutralization area under the curve (AUC) values plotted against participant birth year. (A) Locally Estimated Scatterplot Smoothing (LOESS) implemented in ggplot2 against baseline (d0) and post-vaccination (d28) neutralizing antibody AUC titers for the 2024-25 IBV vaccine (B/Austria/1359417/2021, C.3 (B/Baltimore/JH-899/2023), C.5.1 (B/Baltimore/JH-547/2024), C.3.1/re (B/Baltimore/JH-1192/2025) and rg-C.3.1/re:N197D (B/Baltimore/JH-1192/2025-background). The dotted line represents the limit of detection (AUC = 1.25). **(B)** AUC titers with LOESS fit by biological sex of participants. Statistical tests for age and sex are reported in Table 2. Only C.3.1/re was statistically significant (p=0.005; Kruskal-Wallis nonparametric test with Bonferroni's correction) by birthyear.

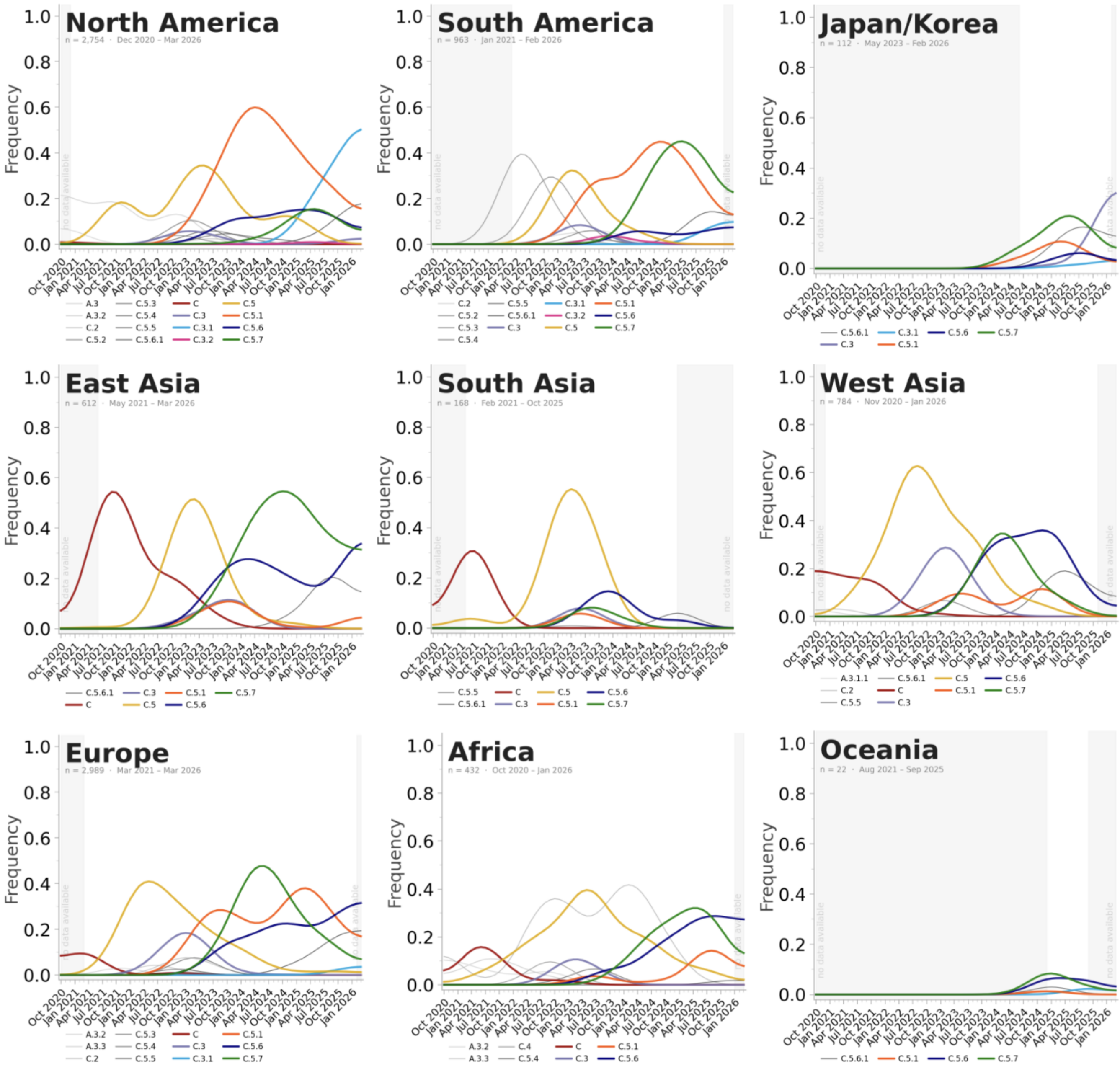

Supplemental Figure S6. Raw global influenza B subclade frequency between October 2020 and March 2026. Ingested sequence data are representative of all HA sequences available in GISAID between October 1, 2020 and March 18, 2026 to visualize relative frequency from all sequences rather than the down selected. Sequence filtering and visualization scripts are available the Quarto notebook:
https://github.com/elginakin/influenzab_c.3/blob/main/notebooks/06_frequencies.qmd

Supplemental Table S1. Total Influenza case counts in the Johns Hopkins Hospital in the 2024-25 season.

DATE	IAV COUNT	IBV COUNT	TOTAL COUNT	IAV %	IBV %
**OCT 2024**	2	0	2	100	0
**NOV 2024**	57	4	61	93.4	6.6
**DEC 2024**	510	12	522	97.7	2.3
**JAN 2025**	1988	41	2029	98	2
**FEB 2025**	2229	65	2294	97.2	2.7
**MAR 2025**	526	372	898	58.6	41.4
**APR 2025**	53	400	453	11.7	88.3
**TOTAL**	5365	894	6259	85.7	14.3

Supplemental Table S2. Clinical and demographic characteristics of patients from the Johns Hopkins Hospital using a sub-cohort of infected individuals with recoverable high-quality IBV whole genome sequences.

	Whole Cohort		C.3		C.5.x	
	count	%N	count	%N	count	%N
**No. of patients**	113		67		46	
**Female**	61	54.00%	39	58.20%	22	47.80%
**Male**	52	46.00%	28	41.80%	24	52.20%
**Age Range**						
**Mean (SD)**	14	13	10.8	8.94	18.6	16.2
**Median**	9		8		13	
**Range**	57		38		57	
**<2**	8	7.10%	<4		<4	
**2-5**	23	20.40%	16	23.90%	7	15.20%
**6-11**	35	31.00%	27	40.30%	8	17.40%
**12-17**	17	15.00%	8	11.90%	9	19.60%
**18-44**	25	22.10%	12	17.90%	13	28.30%
**45-55**	<4		0	0.00%	<4	8.70%
**56-70**	<4		0	0.00%	<4	
**70+**	0	0.00%	0	0.00%	0	0.00%
**Comorbidities**						
**Hypertension**	18	15.90%	9	13.40%	9	19.60%
**Lung Disease**	39	34.50%	21	31.30%	18	39.10%
**Kidney Disease**	18	15.90%	12	17.90%	6	13.00%
**Immunosuppression**	24	21.20%	11	16.40%	13	28.30%
**Diabetes**	9	8.00%	<4		6	13.00%
**Heart failure**	6	5.30%	<4		<4	
**Smoker**	8	7.10%	5	7.50%	<4	
**Cancer**	34	30.10%	23	34.30%	11	23.90%
**Pregnant**	<4	0.90%	0	0.00%	<4	
**Outcome**						
**Admitted**	20	17.70%	12	17.90%	8	17.40%
**ICU**	<4		<4		<4	
**Supplemental Oxygen**	10	8.80%	<4		6	13.00%
**Death**	<4		0	0.00%	<4	
**Symptoms**						
**Chart count**	102		59		43	
**Fever**	32	31.40%	21	35.60%	11	25.60%
**Cough**	10	9.80%	10	16.90%	<4	
**Headache**	<4		<4		<4	
**Breathing problems**	<4		<4		0	0.00%
**Chest pain**	7	6.90%	<4		5	11.60%
**Sore throat**	5	4.90%	<4		<4	
**URI**	6	5.90%	<4		<4	
**Abdominal pain**	9	8.80%	5	8.50%	<4	
**Emesis**	7	6.90%	5	8.50%	<4	
**Flu-like symptoms**	32	31.40%	18	30.50%	14	32.60%
**Weakness**	<4		<4		0	0.00%
**Seizures**	<4		<4		<4	

Supplemental Table S3. Viruses used in this study

Strain Name	HA Clade	HA Subclade	Genotype	Total N-Linked Glycosylation Motifs	GISAID Accession
B/Baltimore/JH-1192/2025	V1A.3a.2	C.3.1	C.3.1/re	11	EPI_ISL_19919784
B/Baltimore/JH-547/2024	V1A.3a.2	C.5.1	N/A	10	EPI_ISL_19861034
B/Baltimore/JH-899/2023	V1A.3a.2	C.3	N/A	10	EPI_ISL_20247894
B/Austria/1359417/2021	V1A.3a.2	C	N/A	10	EPI_ISL_983345
rg-B/Baltimore/JH-1192/2025:[N197D]	V1A.3a.2	C.3.1	C.3.1/re	10	

Supplemental Table S4. 2024-25 JH-CEIRR influenza vaccine cohort age and sex study design

Age Group (Years)	n(Female/Male)
21-29	10 (5/5)
30-39	10 (5/5)
40-49	10 (5/5)
50-59	10 (5/5)
60-69	10 (5/5)
Total	50 (25/25)

Supplemental Table S5. JH-CEIRR influenza vaccine cohort with demographics, Neutralization Titer 50% and Area Under the Curve Values (AUC) by patient.

B/Victoria 2024-25 Vaccine				B/Victoria C.3				B/Victoria C.5.1				B/Victoria C.3.1/re				B/Victoria rg-C.3.1/re:N197D			
B/Austria/1359417/2021-Egg				B/Baltimore/JH-899/2023				B/Baltimore/JH-547/2024				B/Baltimore/JH-1192/2025				B/Baltimore/JH-1192/2025:N197D			
Day 0		Day 28		Day 0		Day 28		Day 0		Day 28		Day 0		Day 28		Day 0		Day 28	
NT50	AUC	NT50	AUC	NT50	AUC	NT50	AUC	NT50	AUC	NT50	AUC	NT50	AUC	NT50	AUC	NT50	AUC	NT50	AUC
320	470	320	710	20	10	80	100	10	0	80	85	10	0	10	0	320	470	320	710
40	50	160	170	40	100	160	130	40	32.5	80	100	10	0	10	0	40	32.5	80	100
5	0	20	27.5	10	0	40	55	10	0	10	0	10	0	10	0	5	0	20	27.5
160	230	160	230	80	130	160	280	160	220	320	457.5	10	0	10	0	160	230	160	230
640	830	640	950	80	100	80	100	80	160	160	190	10	0	10	0	80	160	160	190
160	230	320	350	80	100	160	190	80	55	80	130	10	0	10	0	80	55	80	130
10	8.75	40	65	40	40	40	40	40	40	40	80	10	0	10	0	40	40	40	80
160	170	160	230	40	85	80	100	40	40	80	100	10	0	10	0	160	170	160	230
40	65	40	110	160	280	320	460	320	460	640	760	10	0	10	0	320	460	640	760
160	230	320	470	160	160	160	220	80	97.5	80	85	10	0	10	0	80	85	80	97.5
20	20	640	950	10	0	320	460	10	0	320	0	10	0	10	0	20	20	640	950
20	16.25	160	230	10	0	80	32.5	10	0	40	40	10	0	10	0	10	0	40	40
160	200	320	470	80	85	80	130	40	55	80	100	10	0	10	0	40	55	160	100
20	20	640	950	10	0	160	220	10	0	160	220	10	0	10	0	10	0	160	220
80	110	320	470	10	0	640	940	20	7.5	640	940	10	0	20	15	80	110	320	470
20	20	80	95	10	0	80	70	10	0	40	40	10	0	10	0	20	20	320	95
5	3.75	640	950	10	0	320	580	10	0	640	820	10	0	10	0	10	0	640	820
80	110	160	290	20	10	160	100	40	22.5	80	127.5	10	0	10	0	80	110	160	290
320	470	320	470	80	62.5	80	85	160	340	320	400	10	0	10	0	320	470	320	470
10	8.75	20	20	80	70	320	400	80	100	320	460	10	0	10	0	10	8.75	20	20
80	110	160	230	20	5	40	7.5	20	10	40	32.5	10	0	10	0	20	10	40	32.5
160	290	320	470	10	0	40	32.5	10	0	40	25	10	0	10	0	160	290	320	470
40	50	40	42.5	20	17.5	640	700	80	100	640	820	10	0	20	7.5	40	50	40	42.5
80	110	80	110	80	17.5	80	62.5	40	40	80	70	10	0	10	0	40	40	80	70
160	260	1280	1430	160	220	640	940	160	280	640	940	10	0	10	0	160	260	1280	1430
40	50	160	230	160	115	160	130	80	130	80	97.5	10	0	10	0	40	50	160	230
5	1.25	160	170	20	7.5	40	47.5	20	17.5	40	55	10	0	10	0	20	17.5	40	55
80	110	160	230	10	0	10	0	10	0	10	2.5	10	0	10	0	10	0	10	2.5
20	20	320	470	20	10	160	130	10	0	160	160	10	0	10	0	10	0	160	160
320	470	320	470	10	0	20	7.5	20	7.5	80	100	10	0	20	10	320	470	320	470
40	65	80	87.5	10	0	40	40	10	2.5	40	40	10	0	10	0	10	2.5	40	40
10	1.25	10	5	10	40	160	55	80	0	80	55	10	0	10	0	80	0	80	55
40	42.5	40	50	10	0	160	220	10	0	320	460	10	0	10	0	10	0	320	460
20	20	40	42.5	320	460	320	460	640	700	640	940	10	2.5	20	17.5	20	20	40	42.5
10	5	80	110	320	580	640	760	1280	1900	1280	2380	160	220	160	220	10	5	80	110
5	1.25	640	950	320	460	320	340	320	415	320	460	10	0	10	0	320	415	320	460
5	1.25	40	80	10	17.5	640	940	80	70	1280	1660	20	5	20	5	80	70	1280	1660
80	110	320	470	10	0	80	100	20	7.5	160	160	10	0	10	0	20	7.5	160	160
20	20	40	50	160	250	160	370	160	190	160	220	40	55	80	85	160	190	160	220
5	1.25	320	410	80	100	320	340	320	460	320	460	20	17.5	40	32.5	5	1.25	320	410
160	230	640	710	40	40	80	85	80	85	80	130	10	2.5	40	42.5	160	230	640	710
160	350	320	470	10	0	10	2.5	10	0	20	7.5	10	0	10	0	160	350	320	470
160	200	320	470	80	100	160	220	320	452.5	320	460	10	0	10	0	160	200	320	470
160	200	320	1070	320	280	320	460	320	460	640	700	20	7.5	40	32.5	160	200	320	1070
320	470	320	470	40	40	40	40	40	40	80	70	10	0	10	0	320	470	320	470
40	65	40	65	40	25	80	55	20	7.5	20	7.5	10	0	10	0	40	65	40	65
5	0	320	470	40	47.5	80	85	80	85	80	100	10	0	10	0	80	85	80	100
40	50	80	110	640	700	640	940	640	820	1280	1900	40	70	40	55	320	820	640	1900
80	110	160	230	40	40	80	85	80	92.5	80	112.5	10	0	10	0	80	92.5	80	112.5
160	230	320	470	320	100	640	400	40	40	160	220	10	0	10	0	40	40	160	220
